# The triglyceride-glucose index as a surrogate marker of insulin resistance for predicting peripheral vascular complications in patients with type 2 diabetes: A retrospective analysis

**DOI:** 10.1097/MD.0000000000048910

**Published:** 2026-05-22

**Authors:** Dong Fan, Xiaoyu Ma, Luyao Zhang, Tao Deng

**Affiliations:** a Department of Endocrinology, Fuyang Women and Children’s Hospital, Anhui, China.

**Keywords:** Dose-Response Relationship, Insulin Resistance, Peripheral Vascular Disease, Restricted Cubic Spline, Triglyceride-Glucose Index, Type 2 Diabetes Mellitus

## Abstract

The TyG index, a simple surrogate marker for insulin resistance, shows promise for predicting vascular complications in T2DM. This study evaluated its utility in predicting peripheral vascular complications and analyzed the dose-response relationship. A retrospective analysis included 186 T2DM patients admitted between April and December 2025. Participants were categorized into a complications group (n = 66) and a no-complications group (n = 120). The TyG index was calculated and divided into quartiles. Multivariate logistic regression identified risk factors. RCS curves assessed the dose-response relationship, and ROC curves evaluated predictive value. The complications group was significantly older with longer diabetes duration. They exhibited higher fasting glucose, triglycerides, TyG index, cystatin C, urea, and creatinine levels (all *P* < .05), but lower HDL-C, albumin, eGFR, and hemoglobin levels (all *P* < .05). Prevalence of peripheral vascular disease increased progressively across TyG quartiles (Q1: 8.7%, Q2: 23.9%, Q3: 41.3%, Q4: 68.1%; *P* < .001). Multivariate analysis, using Q1 as reference, showed significantly elevated risks for complications: Q2 (OR = 2.24, 95% CI: 1.02–1.58, *P* = .046), Q3 (OR = 2.89, 95% CI: 1.34–4.31, *P* < .001), and Q4 (OR = 4.47, 95% CI: 2.42–6.58, *P* < .001). RCS analysis confirmed a significant nonlinear positive correlation between TyG index and complication risk (*P* for nonlinearity < .001). ROC analysis demonstrated the TyG index predicted complications with an AUC of 0.768 (95% CI: 0.698–0.838). The optimal cutoff value was 8.89, yielding 75.8% sensitivity and 70.8% specificity. The TyG index exhibits a significant nonlinear positive cross-sectional association with peripheral vascular complications in T2DM patients. While these findings suggest the potential utility of the TyG index as a marker for identifying existing peripheral vascular complications, larger multicenter prospective studies with external validation are needed before clinical implementation can be recommended.

## 1. Introduction

T2DM is a metabolic disorder characterized by chronic hyperglycemia, with its prevalence steadily increasing worldwide. According to the International Diabetes Federation, approximately 537 million adults had diabetes in 2019, and this number is projected to reach 783 million by 2045.^[1]^ China has the largest number of individuals with diabetes, with approximately 141 million cases in 2021, imposing significant economic burdens on society and families.^[2]^

PVD is one of the major chronic complications of T2DM, with an incidence rate of 20%–30% among diabetic patients. PVD significantly impairs quality of life, worsens prognosis, and increases the risk of amputation and mortality.^[3,4]^ The pathogenesis of diabetic peripheral vascular disease is complex, involving multiple mechanisms including dysregulation of glucose and lipid metabolism, chronic inflammation, oxidative stress, and endothelial dysfunction.^[5]^ Early identification of high-risk patients and timely intervention are crucial for improving clinical outcomes.

Chronic inflammation is a hallmark of T2DM and plays a critical role in the development of both T2DM and its vascular complications.^[6,7]^ Clinical and biological risk factors associated with inflammation in patients with type 2 diabetes mellitus include elevated levels of inflammatory cytokines such as tumor necrosis factor-alpha, interleukin-6, and CRP, all of which contribute to endothelial dysfunction and atherosclerosis.^[6]^ Furthermore, pancreatic β-cell dysfunction in type 2 diabetes is intimately linked to inflammation and oxidative stress, both of which exacerbate systemic inflammatory responses and metabolic dysregulation, thereby promoting the development of vascular complications.^[7]^ This inflammatory milieu not only impairs insulin secretion but also enhances insulin resistance, creating a vicious cycle that accelerates the development of both microvascular and macrovascular complications.

Insulin resistance is a central component of the pathogenesis of T2DM and a key pathological basis for the development of vascular complications. It not only disrupts glucose metabolism but also induces lipid metabolism disturbances, activates inflammatory responses, and impairs endothelial function, ultimately promoting the development of atherosclerosis.^[5,8]^ Traditional methods for assessing insulin resistance include the hyperinsulinemic-euglycemic clamp technique, considered the gold standard, and the Homeostasis Model Assessment of Insulin Resistance. However, the clamp method is complex, time-consuming, costly, and difficult to implement widely in clinical practice.^[9]^

In recent years, the triglyceride-glucose index (TyG index) has attracted widespread attention as a simple and practical surrogate marker for insulin resistance. The TyG index was first proposed by Simental-Mendía in 2008. Its calculation formula is: ln [fasting triglycerides (mg/dL) × fasting blood glucose (mg/dL)/2], which requires only routine fasting blood glucose and triglyceride measurements. This method is convenient, cost-effective, and reproducible.^[10]^ Multiple studies have demonstrated that the TyG index correlates well with the hyperinsulinemic-euglycemic clamp and HOMA-IR, effectively reflecting the degree of insulin resistance.^[11]^ Additionally, previous research has shown that the TyG index is closely associated with various cardiovascular risk factors and clinical outcomes, including hypertension, coronary artery disease, stroke, and diabetic nephropathy.^[12–15]^

Peripheral vascular disease is characterized by chronic inflammation. Higher systemic immune-inflammation index is associated with a higher likelihood of peripheral arterial disease,^[16]^ and the role of inflammation in cardiovascular disease is well-established.^[17]^ This inflammation-mediated mechanism represents a critical link between metabolic dysregulation and macrovascular complications in diabetic patients. The convergence of elevated inflammatory markers and vascular dysfunction underscores the importance of investigating biomarkers that reflect both metabolic dysregulation and inflammatory status.

The TyG index has been demonstrated to be associated with inflammatory diseases and adverse cardiovascular outcomes. Recent evidence shows that the predictive value of the triglyceride-glucose index combined with inflammatory markers is superior for major adverse cardiovascular events after percutaneous coronary intervention.^[18]^ Furthermore, large-scale epidemiological studies have revealed an association between the triglyceride-glucose index, body mass index, dietary inflammatory index, and the development of chronic kidney disease in the 1999–2018 National Health and Nutrition Examination Survey cohort.^[19]^ These findings indicate that the TyG index, as a composite marker integrating both glycemic and lipid metabolic information, provides a comprehensive reflection of metabolic dysregulation that impacts systemic inflammation and multiple organ systems.

However, there are relatively few studies investigating the predictive value of the TyG index for peripheral vascular complications in T2DM, particularly regarding the underlying mechanisms linking the TyG index to inflammation and PVD, as well as its dose-response relationship. The specific mechanisms by which insulin resistance, as reflected by elevated TyG index, promotes chronic inflammation and subsequently accelerates PVD development remain underexplored. Understanding these pathophysiological relationships would strengthen the scientific rationale for using the TyG index as a clinical predictor of PVD risk.

RCS is a flexible statistical method capable of identifying nonlinear relationships between continuous variables and outcomes, providing more precise risk assessments for clinical decision-making.^[20]^ Currently, there is a lack of research analyzing the dose-response relationship between the TyG index and diabetic peripheral vascular complications using RCS, or comprehensively examining how the TyG index relates to inflammation-driven vascular disease in T2DM patients.

Therefore, studying the TyG index in association with type 2 diabetes mellitus and peripheral vascular disease is scientifically reasonable and clinically justified. The accumulating evidence linking the TyG index to inflammation, T2DM pathophysiology, and cardiovascular complications provides strong biological plausibility for this investigation. Accordingly, this study aims to explore the clinical utility of the TyG index in predicting peripheral vascular complications in patients with T2DM, employing quartile-based analysis and RCS curves to evaluate its dose-response relationship. The findings will offer scientific support for early high-risk patient identification, illuminate the inflammation-mediated mechanisms linking metabolic dysregulation to PVD development, and facilitate personalized treatment planning.

## 2. Materials and methods

### 2.1. Study design and subjects

This study employed a retrospective cross-sectional design, collecting clinical data from patients with type 2 diabetes mellitus who were hospitalized and treated in the Department of Endocrinology at our hospital from April 2025 to December 2025. Inclusion criteria were as follows: diagnosis of type 2 diabetes mellitus according to the World Health Organization standards from February 2019 defined as fasting blood glucose ≥ 7.0 mmol/L, random blood glucose ≥ 11.1 mmol/L, 2-hour plasma glucose ≥ 11.1 mmol/L during an oral glucose tolerance test, or HbA1c ≥ 6.5%; age ≥ 18 years; completion of relevant examinations during hospitalization with complete clinical data; and signed informed consent. Additional eligibility requirements included: clinical stability at the time of laboratory assessment, defined as hemodynamically stable condition without requirement for intensive monitoring or acute interventions, and absence of significant acute physiological stress as evidenced by normal or near-normal vital signs and absence of acute inflammatory markers. The exclusion criteria included: type 1 diabetes, gestational diabetes, or other specific types of diabetes; acute diabetic complications such as diabetic ketoacidosis or hyperosmolar hyperglycemic state; malignant tumors, severe cardiac insufficiency (NYHA class III-IV), severe hepatic impairment (Child-Pugh class C), or end-stage renal disease requiring dialysis; acute infections, trauma, or surgery within the past month; use of immunosuppressants or corticosteroids; severe psychiatric disorders or cognitive impairment; and incomplete clinical data or patients lost to follow-up. Following screening through the hospital information system based on these inclusion and exclusion criteria, a total of 186 eligible patients with type 2 diabetes were finally included in the study. This research was approved by the hospital’s Ethics Committee, and all patients provided written informed consent.

### 2.2. Definition and diagnostic criteria for peripheral vascular disease

PVD was defined using a comprehensive approach that combines functional, anatomical, and clinical criteria to ensure sensitive detection of vascular pathology across the disease spectrum. PVD was diagnosed when patients met any of the following criteria: ABI <0.9, indicating hemodynamically significant disease;  ≥ 50% stenosis of lower limb arteries observed on duplex ultrasonography; confirmed ≥ 50% stenosis of lower limb arteries by lower limb angiography or CTA; or presence of characteristic intermittent claudication symptoms supported by Doppler ultrasonography or imaging studies. Standardized evaluation protocol: All patients underwent systematic vascular assessment according to a predefined protocol. Universal screening included ABI measurement for all participants. Subsequent imaging studies (duplex ultrasonography, CTA, or angiography) were performed based on specific clinical indications: ABI < 0.9 or > 1.3 (suggesting vascular calcification), clinical symptoms suggestive of peripheral vascular disease (intermittent claudication, rest pain, or nonhealing wounds), physical examination findings indicating vascular insufficiency (absent pulses, bruits, or delayed capillary refill), or high clinical suspicion based on diabetes duration > 10 years combined with other cardiovascular risk factors. Rationale for composite definition: This multi-modal diagnostic approach was designed to capture the full spectrum of peripheral vascular disease, from subclinical functional impairment to advanced anatomical stenosis. The combination of functional (ABI) and anatomical (imaging) criteria addresses the limitations of individual diagnostic methods, particularly in diabetic patients where vessel calcification may affect ABI accuracy. The inclusion of both symptomatic and asymptomatic disease reflects the clinical reality that early intervention for subclinical disease can prevent progression to symptomatic stages.

To minimize verification bias and ensure diagnostic consistency, the following quality control measures were implemented: Standardized ABI measurement: All ABI measurements were performed by trained technicians using standardized protocols with patients in supine position after 10 minutes of rest; Imaging quality assurance: All vascular imaging studies were performed by experienced sonographers and interpreted by qualified radiologists blinded to patient TyG index values; Clinical assessment standardization: Symptoms were assessed using standardized questionnaires for intermittent claudication (Edinburgh Claudication Questionnaire) and documented according to Fontaine classification; Multi-reviewer consensus: Borderline cases underwent review by 2 independent clinicians to ensure diagnostic accuracy; Documentation of imaging rationale: Clear documentation was maintained for all patients regarding the clinical indication for advanced imaging studies to enable assessment of potential selection bias.

### 2.3. Clinical data collection

#### 2.3.1. Baseline clinical data

Baseline clinical data of the patients were collected through the electronic medical record system, including the following: Demographic characteristics: age (recorded as actual age), and gender; Physical examination: BMI, calculated as weight (kg) divided by height squared (m^2^); Medical history: duration of diabetes (calculated from the date of diagnosis to the present, accurate to the month), and history of hypertension (defined as previously diagnosed hypertension or systolic blood pressure ≥ 140 mm Hg and/or diastolic blood pressure ≥ 90 mm Hg); Medication history: use of hypoglycemic agents (including insulin, metformin, etc).

#### 2.3.2. Laboratory test indicators

All patients had venous blood samples collected after an overnight fast of 12 hours on the morning following admission. To minimize the potential impact of acute hospitalization stress on metabolic measurements, blood collection was performed at least 24 hours after admission when patients had achieved clinical stability, defined as absence of acute symptoms, stable vital signs, and absence of immediate metabolic interventions. The blood samples were analyzed according to the standardized procedures of our hospital’s laboratory. All tests were completed within 2 hours of blood collection, using uniform testing equipment and reagents. Patients were instructed to maintain their usual dietary patterns prior to the fasting period, and any recent changes in lipid-lowering medications or acute metabolic interventions were documented and considered as potential confounding factors. The collected laboratory indicators included: Blood glucose metabolism markers: FPG(mmol/L) and HbA1c; Lipid profile: TC(mmol/L), TG(mmol/L), HDL-C(mmol/L) and LDL-C(mmol/L); Protein markers: ALB(g/L), GLB(g/L), and ACR(mg/g); Renal function indicators: Cys-C(mg/L), BUN(mmol/L), serum creatinine(μmol/L), UA(μmol/L), and eGFR(mL/min/1.73m^2^); Liver enzymes: LDH(U/L) and α-HBDH(U/L); Routine blood parameters: NEU(×10^9^/L), LYM(×10^9^/L), MONO(×10^9^/L), Hb(g/L), and PLT(×10^9^/L). Computed index: TyG index, calculated as ln[TG(mg/dL) × FPG(mg/dL)/2], where conversion factors are applied: TG(mmol/L) × 88.57, FPG(mmol/L) × 18.02.

### 2.4. TyG index quartile grouping

All patients were ranked according to their TyG index levels from lowest to highest and divided into 4 quartile groups based on the distribution: Q1 group: TyG index ≤ 25th percentile; Q2 group: 25th percentile < TyG index ≤ 50th percentile; Q3 group: 50th percentile < TyG index ≤ 75th percentile; Q4 group: TyG index > 75th percentile.

### 2.5. Quality control

To ensure data quality and the reliability of the study, the following quality control measures were implemented: All personnel involved in data collection received standardized training to familiarize themselves with the study protocol and data collection standards. A standardized case report form(CRF) was developed, and data entry was performed by 2 individuals independently, followed by cross-verification to ensure accuracy. Values exceeding 3 times the standard deviation from the normal range were rechecked; only confirmed data were included in the analysis. For variables with <5% missing data, complete case analysis was used; for variables with missing data of 5% or more, multiple imputation methods were applied. Additionally, periodic data quality assessments were conducted to identify and correct any issues in data collection in a timely manner. To address potential measurement bias from acute hospitalization effects, additional quality control measures were implemented: (1) Clinical stability assessment: patients were evaluated for hemodynamic stability and absence of acute metabolic derangements before blood collection; (2) Medication review: any recent changes in diabetes medications, lipid-lowering therapy, or corticosteroid use within 72 hours of admission were documented; (3) Dietary assessment: patients with reported significant dietary changes or poor oral intake in the 48 hours prior to admission were identified for sensitivity analysis; (4) Stress marker evaluation: patients with evidence of significant physiological stress (elevated C-reactive protein > 10 mg/L, leukocytosis > 15,000/μL, or fever > 38°C) at the time of blood collection were flagged for separate analysis.

### 2.6. Statistical methods

Statistical analyses were performed using SPSS version 26.0 and R version 4.3.0. Normality of continuous variables was assessed using the Shapiro–Wilk and Kolmogorov-Smirnov tests. Normally distributed data are expressed as means ± standard deviations(x̄±s), while non-normally distributed data are presented as medians with interquartile ranges[M(Q1, Q3)]. Categorical variables are reported as counts(percentages)[n(%)]. For between-group comparisons: normally distributed continuous variables were compared using independent sample t-tests or one-way ANOVA, while non-normally distributed variables were analyzed with the Mann–Whitney U test or Kruskal-Wallis H test. Categorical variables were compared using chi-square tests or Fisher’s exact test. Multiple pairwise comparisons among groups were conducted with Bonferroni correction.

The correlation between the TyG index and various variables was evaluated using Pearson’s correlation coefficient or Spearman’s rank correlation coefficient, as appropriate. The Cochran-Armitage trend test was applied to assess the trend in peripheral vascular disease prevalence across TyG quartiles. Variables with *P*-values <.05 in univariate analyses were included in multivariate logistic regression models. Stepwise variable selection was performed using the forward likelihood ratio (Forward LR) method to identify independent risk factors. To prevent collinearity, variance inflation factors were calculated for the variables; those with VIF > 10 were excluded from the model. Receiver operating characteristic curves were plotted, and the area under the curve with 95% confidence intervals was calculated. The DeLong test was used to compare the predictive performance of different models. The optimal cutoff point was determined using the Youden index, from which sensitivity, specificity, positive predictive value, negative predictive value, positive likelihood ratio, and negative likelihood ratio were derived. Restricted cubic spline regression analysis was conducted to explore the dose-response relationship between the TyG index and the risk of peripheral vascular complications, with 4 knots at the 5th, 35th, 65th, and 95th percentiles, using the 10th percentile of the TyG index as the reference value. The statistical significance of any nonlinear relationship was evaluated.

All tests were two-sided, with *P* < .05 indicating statistical significance. Considering multiple comparisons, the Bonferroni correction was applied for pairwise comparisons among quartile.

## 3. Results

### 3.1. Baseline characteristics of study subjects

This study enrolled a total of 186 patients with type 2 diabetes mellitus, including 102 males (54.8%) and 84 females (45.2%), with an average age of 62.4 ± 11.2 years. The duration of diabetes ranged from 0.5 to 32 years, with a median duration of 10.2 years. Among the participants, 66 patients (35.5%) had peripheral vascular disease, while 120 patients (64.5%) did not.

The mean TyG index was 8.6 ± 0.7. Participants were divided into 4 quartile groups: Q1 (n = 47): TyG index ≤ 8.2; Q2 (n = 46): 8.2 < TyG index ≤ 8.7; Q3 (n = 46): 8.7 < TyG index ≤ 9.3; Q4 (n = 47): TyG index > 9.3. The baseline clinical characteristics of the 2 patient groups are summarized in Table 1. Patients with complications were significantly older than those without [(66.8 ± 9.8) vs (59.7 ± 11.4) years, *P* < .001], had a longer duration of diabetes [(12.6 ± 7.4) vs (8.9 ± 6.2) years, *P* < .001], and a higher prevalence of hypertension [75.8% vs 55.0%, *P* = .006]. There were no significant differences between the 2 groups in terms of gender or BMI (*P* > .05).

**Table 1:** Comparison of baseline clinical characteristics between the 2 groups of patients.

Variables	Total (n = 186)	Non-PVD group (n = 120)	PVD group (n = 66)	*P*-Value
**Age (Years**)	62.4 ± 11.2	59.7 ± 11.4	66.8 ± 9.8	<.001
**Gender [n(%)]**				.534
Male	102 (54.8)	64 (53.3)	38 (57.6)	
Female	84 (45.2)	56 (46.7)	28 (42.4)	
**BMI (kg/m**^**2**^)	24.7 ± 3.6	24.9 ± 3.8	24.3 ± 3.2	.277
**Diabetes duration (Years**)	10.3 ± 7.1	8.9 ± 6.2	12.6 ± 7.4	<.001
**Hypertension [n(%)]**	116 (62.4)	66 (55.0)	50 (75.8)	.006
**Antidiabetic drug use [n(%)]**				
Insulin	128 (68.8)	78 (65.0)	50 (75.8)	.140
Metformin	142 (76.3)	94 (78.3)	48 (72.7)	.399

BMI = body mass index.

### 3.2. Comparison of laboratory parameters

The laboratory indicators for the 2 patient groups are summarized in Table 2. In terms of glucose metabolism, patients in the complication group exhibited significantly higher fasting blood glucose levels [(9.8 ± 3.2) mmol/L] compared to the non-complication group [(8.4 ± 2.6) mmol/L] (*P* = .002). Although the HbA1c level showed an upward trend in the complication group [(9.3 ± 2.1)%] vs [(8.9 ± 1.8)%], this difference was not statistically significant (*P* = .173). Regarding lipid metabolism, the complication group demonstrated significantly elevated triglyceride levels [(2.4 ± 1.8) mmol/L] vs [(1.7 ± 1.1) mmol/L] (*P* = .003), and significantly decreased HDL-C levels [(1.0 ± 0.3) mmol/L] compared to [(1.2 ± 0.4) mmol/L] (*P* = .001). There were no significant differences in total cholesterol and LDL-C levels between the groups. In renal function parameters, patients in the complication group showed significantly higher levels of cystatin C [(1.4 ± 0.5) mg/L] vs [(1.1 ± 0.3) mg/L] (*P* < .001), blood urea nitrogen [(7.8 ± 3.2) mmol/L] vs [(6.2 ± 2.1) mmol/L] (*P* = .001), and serum creatinine [(89.6 ± 28.4) μmol/L] vs [(76.3 ± 19.2) μmol/L] (*P* = .001). Additionally, their estimated glomerular filtration rate (eGFR) was significantly lower [(78.6 ± 24.3) mL/min/1.73 m^2^] compared to [(89.2 ± 20.1) mL/min/1.73 m^2^] (*P* = .003). The ACR was also significantly higher in the complication group. Furthermore, the TyG index was significantly elevated in patients with complications [(9.2 ± 0.8)] relative to those without [(8.6 ± 0.7)] (*P* < .001), representing a key finding of this study.

**Table 2:** Comparison of laboratory indicators between the 2 groups of patients.

Variables	Non-PVD group (n = 120)	PVD group (n = 66)	*P*-Value
**Blood glucose metabolism indicators**			
FPG(mmol/L)	8.4 ± 2.6	9.8 ± 3.2	.002
HbA1c(%)	8.9 ± 1.8	9.3 ± 2.1	.173
**Lipid parameters**			
TC(mmol/L)	4.8 ± 1.2	4.9 ± 1.4	.609
TG(mmol/L)	1.7 ± 1.1	2.4 ± 1.8	.003
HDL-C(mmol/L)	1.2 ± 0.4	1.0 ± 0.3	.001
LDL-C(mmol/L)	2.9 ± 0.9	3.1 ± 1.1	.219
**Protein index**			
Albumin(g/L)	40.2 ± 4.8	37.8 ± 5.6	.003
Globulin(g/L)	29.1 ± 4.2	30.4 ± 5.1	.070
ACR(mg/g)	45.6 (18.2, 89.3)	78.4 (32.6156.7)	.003
**Renal function indicators**			
Cystatin C(mg/L)	1.1 ± 0.3	1.4 ± 0.5	<.001
Urea(mmol/L)	6.2 ± 2.1	7.8 ± 3.2	<.001
Creatinine(μmol/L)	76.3 ± 19.2	89.6 ± 28.4	.001
Uric acid(μmol/L)	312.4 ± 89.6	334.7 ± 98.2	.119
eGFR(mL/min/1.73m^2^)	89.2 ± 20.1	78.6 ± 24.3	.003
**Liver enzyme indicators**			
LDH(U/L)	189.3 ± 45.6	198.7 ± 52.1	.215
α-HBDH(U/L)	145.2 ± 38.9	152.8 ± 44.2	.236
**Blood routine indicators**			
Neutrophils(×10^9^/L)	4.2 ± 1.8	4.8 ± 2.1	.048
Lymphocytes(×10^9^/L)	1.9 ± 0.7	1.8 ± 0.6	.308
Monocyte(×10^9^/L)	0.4 ± 0.2	0.5 ± 0.2	.147
Hemoglobin(g/L)	128.4 ± 18.6	121.2 ± 20.8	.018
Platelets(×10^9^/L)	245.7 ± 78.2	258.9 ± 85.6	.302
**Calculated Indices**			
TyG Index	8.6 ± 0.7	9.2 ± 0.8	<.001

ACR = urine microalbumin-to-creatinine ratio, eGFR = estimated glomerular filtration rate, FPG = fasting plasma glucose, HbA1c = glycated hemoglobin, HDL-C = high-density lipoprotein cholesterol, LDH = lactate dehydrogenase, LDL-C = low-density lipoprotein cholesterol, TC = total cholesterol, TG = triglycerides, TyG = triglyceride-Glucose, α-HBDH = α-hydroxybutyrate dehydrogenase.

### 3.3. TyG index quartile grouping analysis

**Figure 1.**
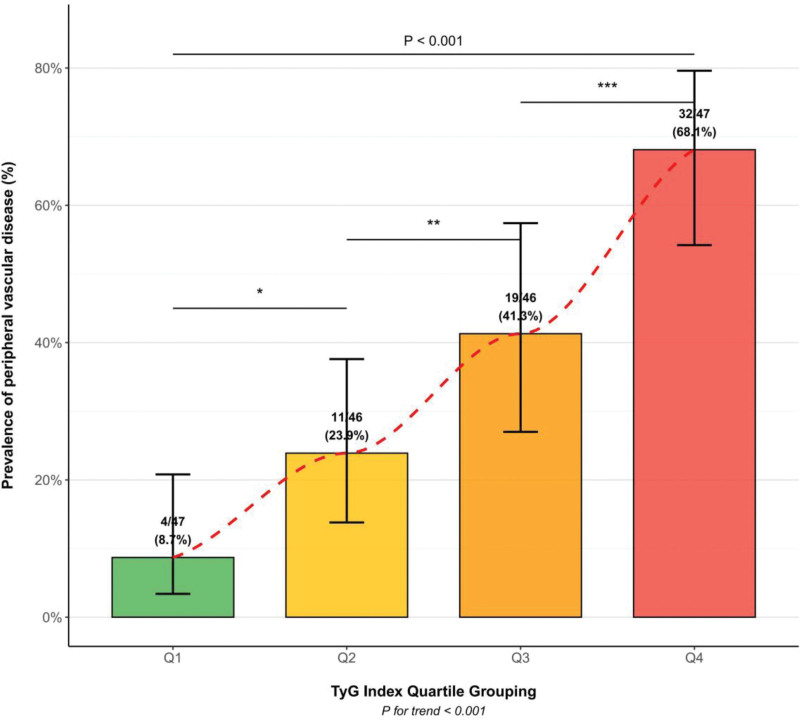
Comparison of peripheral vascular lesion prevalence across TyG index quartiles. TyG = triglyceride-glucose.

The comparison of clinical characteristics among patients divided into quartiles based on the TyG index is presented in Table 3. The TyG index ranges for each group were as follows: Q1 < 8.2; Q2 ≥ 8.2 and < 8.7; Q3 ≥ 8.7 and < 9.3; Q4 ≥ 9.3. As the TyG index quartile increased, there was a gradual elevation in patient age, duration of diabetes, fasting blood glucose, triglycerides, cystatin C, and serum creatinine (*P* < .05). Conversely, levels of HDL-C, albumin, and eGFR showed a decreasing trend with increasing TyG quartile (*P* < .05). The most significant finding was the markedly increasing prevalence of peripheral vascular disease across TyG quartiles: 4 cases (8.7%) in Q1, 11 cases (23.9%) in Q2, 19 cases (41.3%) in Q3, and 32 cases (68.1%) in Q4, with a trend *P*-value < .001 (Fig. 1).

**Table 3:** Comparison of clinical characteristics among patients grouped by TyG index quartiles.

Variables	Q1 (n = 47)	Q2 (n = 46)	Q3 (n = 46)	Q4 (n = 47)	*P*-Value	*P* for Trend
**TyG Index**	7.8 ± 0.3	8.5 ± 0.1	9.0 ± 0.2	9.8 ± 0.4	<.001	<.001
**Age(Years**)	56.8 ± 12.1	60.3 ± 11.2	64.8 ± 10.4	67.5 ± 8.9	<.001	<.001
**Female [n(%)]**	24 (51.1)	25 (54.3)	26 (56.5)	27 (57.4)	.906	.534
**BMI (kg/m**^**2**^)	23.9 ± 3.2	24.7 ± 3.6	25.1 ± 3.8	25.2 ± 3.9	.133	.045
**Diabetes duration (Years**)	7.2 ± 5.8	9.1 ± 6.4	11.6 ± 7.3	13.4 ± 7.9	<.001	<.001
**Hypertension [n(%)]**	22 (46.8)	26 (56.5)	32 (69.6)	36 (76.6)	.039	.002
**FPG (mmol/L**)	6.8 ± 1.9	8.2 ± 2.3	9.4 ± 2.8	11.2 ± 3.4	<.001	<.001
**HbA1c (%**)	8.4 ± 1.6	8.9 ± 1.9	9.2 ± 2.0	9.6 ± 2.1	.007	.002
**TG (mmol/L**)	1.1 ± 0.4	1.5 ± 0.7	2.1 ± 1.2	3.2 ± 2.1	<.001	<.001
**HDL-C (mmol/L**)	1.3 ± 0.4	1.2 ± 0.4	1.1 ± 0.3	0.9 ± 0.3	<.001	<.001
**Cystatin C (mg/L**)	1.0 ± 0.2	1.1 ± 0.3	1.3 ± 0.4	1.5 ± 0.6	<.001	<.001
**Creatinine (μmol/L**)	69.8 ± 15.4	75.2 ± 19.8	82.6 ± 24.1	94.3 ± 31.2	<.001	<.001
**eGFR (mL/min/1.73m**^**2**^)	95.6 ± 18.2	88.4 ± 20.5	83.7 ± 22.8	76.2 ± 25.1	<.001	<.001
**Abbumin (g/L**)	41.2 ± 4.2	40.1 ± 5.1	38.9 ± 5.4	37.1 ± 5.8	<.001	<.001
**Hemoglobin (g/L**)	130.2 ± 17.8	127.8 ± 19.2	125.1 ± 20.4	121.6 ± 20.9	.075	.012
**PVD [n(%)]**	4 (8.7)	11 (23.9)	19 (41.3)	32 (68.1)	<.001	<.001

eGFR = estimated glomerular filtration rate, FPG = fasting plasma glucose, HbA1c = glycated hemoglobin, HDL-C = high-density lipoprotein cholesterol, PVD = peripheral vascular disease, TG = triglycerides, TyG = triglyceride-glucose.

### 3.4. Analysis of correlation between TyG index and each variable

The results of the correlation analysis between the TyG index and various clinical and laboratory parameters are summarized in Table 4. The TyG index was significantly positively correlated with age (*R* = 0.342, *P* < .001), duration of diabetes (*R* = 0.298, *P* < .001), fasting blood glucose (*R* = 0.723, *P* < .001), triglycerides (*R* = 0.856, *P* < .001), cystatin C (*R* = 0.287, *P* < .001), blood urea nitrogen (*R* = 0.245, *P* = .001), and serum creatinine (*R* = 0.234, *P* = .002). Conversely, it was significantly negatively correlated with HDL-C (r = −0.398, *P* < .001), albumin (r = −0.223, *P* = .002), eGFR (r = −0.267, *P* < .001), and hemoglobin (r = −0.189, *P* = .011).

**Table 4:** Correlation analysis between the TyG index and various variables.

Variables	Correlation coefficient (r)	*P*-Value
**Age**	0.342	<.001
**Diabetes duration**	0.298	<.001
**BMI**	0.087	.236
**FPG**	0.723	<.001
**HbA1c**	0.156	.034
**TG**	0.856	<.001
**HDL-C**	−0.398	<.001
**Albumin**	−0.223	.002
**Cystatin C**	0.287	<.001
**Urea**	0.245	.001
**Creatinine**	0.234	.002
**eGFR**	−0.267	<0.001
**Hemoglobin**	−0.189	0.011
**Neutrophils**	0.178	0.016

BMI = Body mass index, eGFR = estimated glomerular filtration rate, FPG = Fasting plasma glucose, HbA1c = glycated hemoglobin, HDL-C = High-density lipoprotein cholesterol, TG = Triglycerides, TyG = Triglyceride-Glucose.

### 3.5. Multivariate analysis of risk factors for peripheral vascular complications

#### 3.5.1. Analysis of TyG index as a continuous variable

Variables with *P*-values < .05 in univariate analysis, including age, duration of diabetes, hypertension, fasting blood glucose, triglycerides, HDL-C, albumin, cystatin C, blood urea nitrogen, serum creatinine, eGFR, hemoglobin, neutrophil count, and the TyG index, were incorporated into the multivariate logistic regression model. To prevent collinearity issues since the TyG index was highly correlated with fasting blood glucose and triglycerides (VIF > 10) only the TyG index was included in the final model, excluding fasting blood glucose and triglycerides. Using the forward likelihood ratio (Forward LR) method for variable selection, the final model results are presented in Table 5. The results indicated that the TyG index (OR = 3.247, 95% CI: 1.856–5.679, *P* < .001) was an independent risk factor for the development of peripheral vascular complications in patients with type 2 diabetes.

**Table 5:** Multivariate logistic regression analysis of risk factors for peripheral vascular complications (TyG index as a continuous variable).

Variables	β	SE	Wald χ^2^	*P*-Value	OR	95%CI
**TyG Index**	1.178	0.283	17.345	<.001	3.247	1.856–5.679
**Age**	0.051	0.014	13.265	<.001	1.053	1.024–1.083
**Diabetes duration**	0.075	0.022	11.578	.001	1.078	1.032–1.126
**Hypertension**	0.642	0.356	3.251	.031	2.100	1.046–3.814
**Cystatin C**	0.798	0.412	3.748	.043	2.222	1.001–4.981
**Constant term**	−12.456	2.687	21.456	<.001	-	-

TyG = Triglyceride-Glucose.

#### 3.5.2. TyG index quartile grouping analysis

Using Q1 as the reference group, the risk of developing peripheral vascular complications across the groups was analyzed; the results are shown in Table 6. The findings indicate that, compared to Q1, the risk of peripheral vascular complications increased progressively in Q2, Q3, and Q4. After adjusting for confounding factors, the OR was 2.24 (95% CI: 1.02–4.58, *P* = .046), 2.89 (95% CI: 1.34–4.31, *P* < .001), and 4.47 (95% CI: 2.42–6.58, *P* < .001), respectively.

**Table 6:** Multivariate logistic regression analysis of TyG index quartile groups in predicting peripheral vascular complications.

TyG Index Group	Crude OR (95%CI)	*P*-Value	Adjusted OR* (95%CI)	*P*-Value
**Q1**	1.00 (References)	-	1.00 (References)	-
**Q2**	1.28 (1.06–1.53)	.039	1.24 (1.02–1.58)	.046
**Q3**	3.23 (1.52–4.75)	<.001	2.89 (1.34–4.31)	<.001
**Q4**	5.33 (3.42–7.31)	<.001	4.47 (2.42–6.58)	<.001

* Adjusted factors: age, duration of diabetes, hypertension, cystatin C; TyG = Triglyceride-Glucose.

### 3.6. ROC curve analysis of TyG index for predicting peripheral vascular complications

**Figure 2.**
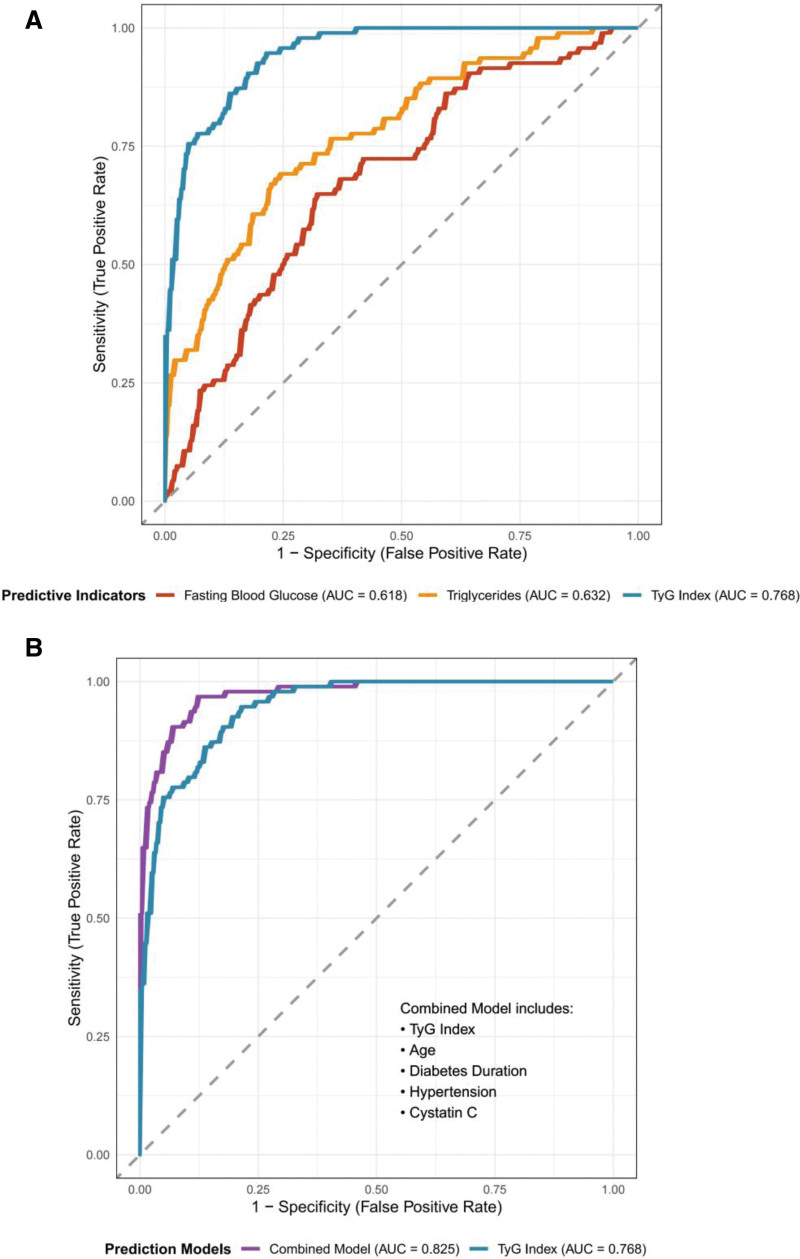
ROC curve for TyG index predicting peripheral vascular complications; (A, B) Comparison of ROC curves between the single indicator, the comprehensive prediction model, and the TyG index. TyG = triglyceride-glucose.

ROC curve analysis revealed that the TyG index discriminated between patients with and without peripheral vascular complications with an area under the curve of 0.768 (95% CI: 0.698–0.838, *P* < .001), indicating good predictive performance. The optimal cutoff value determined by the Youden index was 8.89, with a corresponding sensitivity of 75.8%, specificity of 70.8%, positive predictive value of 58.8%, negative predictive value of 83.7%, positive likelihood ratio of 2.60, and negative likelihood ratio of 0.34. Compared to other individual indicators, the TyG index showed superior predictive value than triglycerides alone (AUC = 0.632, *P* = .018) and fasting blood glucose alone (AUC = 0.618, *P* = .026) (Fig. 2A). A comprehensive predictive model incorporating the TyG index, age, duration of diabetes, hypertension, and cystatin C was constructed, achieving an AUC of 0.825 (95% CI: 0.762–0.888, *P* < .001), which was significantly superior to the model using the TyG index alone (DeLong’s test, *P* = .023) (Fig. 2B).

### 3.7. Restrictive cubic spline curve analysis

**Figure 3.**
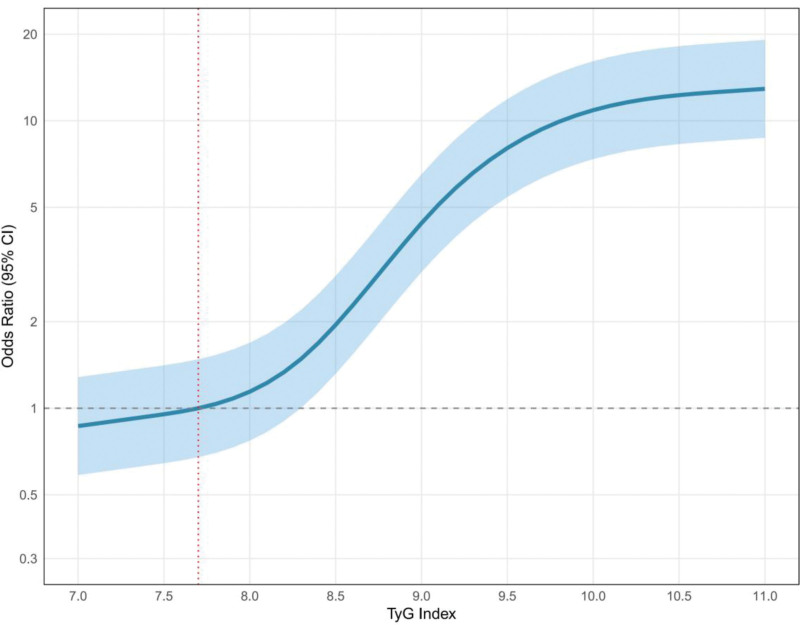
Restricted cubic spline curve showing the dose-response relationship between the TyG index and the risk of peripheral vascular lesions. TyG = triglyceride-glucose.

RCS regression analysis was employed to examine the dose-response relationship between the TyG index and the risk of peripheral vascular complications. Four knots were selected at the 5th, 35th, 65th, and 95th percentiles (corresponding to TyG index values of 7.4, 8.3, 9.1, and 10.8), using the 10th percentile (TyG index = 7.7) as the reference. The RCS analysis revealed a nonlinear positive association between the TyG index and the risk of peripheral vascular complications (nonlinear *P* < .001). Specifically, the risk remained relatively low and stable within the low TyG index range (<8.0); began to increase significantly and at a gradually accelerating rate in the moderate range (8.0–9.0); then increased sharply and exponentially in the high range (>9.0); and finally, the rate of increase started to plateau at very high levels (>10.0), although the risk remained high (Fig. 3).

### 3.8. Stratified analysis

To evaluate the predictive value of the TyG index across different subgroup populations, a stratified analysis was conducted, and the results are shown in Table 7. The findings indicate that the TyG index demonstrated good predictive performance in all subgroups. Notably, the predictive effect was relatively stronger in subgroups of patients aged ≥ 65 years, with a duration of diabetes <10 years, absence of hypertension, and BMI < 25 kg/m^2^.

**Table 7:** The predictive performance of the TyG index for peripheral vascular complications across different subgroups.

Stratified variables	Subgroup	AUC (95%CI)	*P*-Value	Cut-Off	Sensitivity (%)	Specificity (%)
**Age**	<65	0.743 (0.648–0.838)	<.001	8.85	72.7	69.8
	≥65	0.789 (0.687–0.891)	<.001	8.92	78.3	73.2
**Gender**	Male	0.761 (0.663–0.858)	<.001	8.87	73.7	71.9
	Female	0.774 (0.665–0.882)	<.001	8.91	78.6	69.6
**Diabetes duration**	<10	0.812 (0.695–0.929)	<.001	8.76	80.0	75.0
	≥10	0.724 (0.626–0.822)	<.001	8.95	71.9	67.3
**Hypertension**	No	0.798 (0.664–0.932)	<.001	8.82	81.2	72.2
	Yes	0.751 (0.660–0.842)	<.001	8.93	72.0	70.2
**BMI**	<25	0.785 (0.690–0.880)	<.001	8.88	76.9	74.1
	≥25	0.746 (0.634–0.858)	<.001	8.90	74.3	68.0

BMI = body mass index, TyG = triglyceride-glucose.

## 4. Discussion

This study systematically evaluated the cross-sectional association between the TyG index and peripheral vascular disease in 186 patients with type 2 diabetes. The findings indicate that the TyG index is independently associated with prevalent peripheral vascular complications, suggesting its potential utility as a biomarker for identifying existing disease rather than predicting future complications.

Methodological considerations regarding PVD diagnosis. Our composite diagnostic approach warrants discussion regarding potential heterogeneity and verification bias. While the use of multiple diagnostic criteria may introduce some heterogeneity, this approach reflects real-world clinical practice where PVD diagnosis requires integration of functional, anatomical, and clinical assessment. The combination of ABI measurement with selective imaging provides a practical balance between diagnostic sensitivity and resource utilization. Although verification bias cannot be entirely eliminated when imaging is performed selectively, our standardized protocol with clear indications for advanced testing minimizes this concern. The inclusion of both symptomatic and asymptomatic disease aligns with current understanding that subclinical peripheral vascular pathology represents an important target for early intervention in diabetic patients.

The functional-anatomical diagnostic approach has several advantages: ABI measurement provides an objective assessment of hemodynamic significance, while imaging studies offer detailed anatomical information about stenosis location and severity. This combination is particularly important in diabetic patients, where medial sclerosis may cause artificially elevated ABI values, potentially masking significant disease. Our finding that TyG index demonstrates consistent predictive performance across this diagnostically diverse population suggests its utility as a screening tool regardless of the specific manifestation of peripheral vascular pathology.

As a convenient and accessible measure of insulin resistance, the TyG index’s clinical utility has garnered increasing attention.^[10]^ Our study found that patients with complications had significantly higher TyG index values compared to those without complications (9.2 ± 0.8 vs 8.6 ± 0.7, *P* < .001), consistent with previous research by Park et al^[21]^ in a Korean population. Insulin resistance plays a central role in the pathogenesis of type 2 diabetes and is also a key underlying mechanism in the development of vascular complications.^[8]^ The TyG index, which integrates fasting blood glucose and triglyceride levels, effectively reflects the body’s insulin sensitivity status.^[9]^ Compared to gold standard methods such as the hyperinsulinemic-euglycemic clamp, the TyG index offers advantages of being noninvasive, cost-effective, and easy to implement, making it more suitable for clinical practice.^[11]^

Our quartile-based analysis showed a significant increasing trend in the prevalence of peripheral vascular disease with rising TyG index levels: 8.7% in Q1, 23.9% in Q2, 41.3% in Q3, and 68.1% in Q4 (*P* for trend < .001). This dose-response relationship was further validated by restricted cubic spline regression analysis, demonstrating a nonlinear positive association between the TyG index and risk of peripheral vascular complications. Similar dose-response relationships were observed in a prospective study by Zhao et al^[22]^ involving 5318 patients with type 2 diabetes. Notably, in the high-risk range where TyG > 9.0, the risk of complications increased exponentially, suggesting that clinicians should consider more aggressive interventions for patients with TyG indices exceeding 9.0.^[23]^

Multivariate logistic regression analysis identified TyG index, age, duration of diabetes, hypertension, and cystatin C as independent risk factors for peripheral vascular complications, a finding consistent with prior studies.^[24]^ Li et al^[19]^ reported that the TyG index was an independent predictor of peripheral vascular disease in a cohort of 2456 patients. The importance of traditional risk factors such as age and diabetes duration was reaffirmed in this study. As a sensitive early marker of renal function impairment, cystatin C’s independent predictive value further underscores the close link between microvascular renal damage and macrovascular complications.

ROC curve analysis revealed that the TyG index predicted peripheral vascular complications with an area under the curve of 0.768, with an optimal cutoff of 8.89, sensitivity of 75.8%, and specificity of 70.8%. These results are comparable to those reported by Wang et al^[25]^ (AUC = 0.752). Significantly, the predictive performance of the TyG index was superior to that of triglycerides alone (AUC = 0.632) and fasting blood glucose alone (AUC = 0.618), supporting its value as a composite marker.^[26]^ Incorporating TyG, age, diabetes duration, hypertension, and cystatin C into a combined predictive model improved the AUC to 0.825, demonstrating the advantage of multifactorial prediction.

The association between the TyG index and vascular disease likely involves multiple pathophysiological mechanisms. Firstly, insulin resistance-induced hyperinsulinemia directly promotes vascular smooth muscle cell proliferation and vessel wall remodeling.^[27]^ Secondly, insulin resistance is often accompanied by chronic inflammation; the elevated neutrophil levels observed in the complication group support this.^[28]^ Inflammatory cytokines such as tumor necrosis factor-alpha and interleukin-6 can activate endothelial cells and facilitate atherosclerosis development.^[29]^ Additionally, insulin resistance is closely linked to oxidative stress, which damages endothelial function and accelerates vascular lesion progression.

The study also observed significantly elevated triglyceride levels and reduced HDL-C levels in patients with complications an atherogenic lipid profile closely associated with insulin resistance. Hypertriglyceridemia not only directly contributes to atherosclerosis but also accelerates vascular damage through mechanisms such as affecting lipoprotein metabolism and promoting the formation of small dense LDL particles.^[30]^ The TyG index, by integrating triglyceride levels, provides a more comprehensive reflection of lipid metabolic disturbances impacting the vasculature.^[31]^

Moreover, patients with complications exhibited worsened renal function, characterized by increased cystatin C, blood urea nitrogen, and serum creatinine levels, alongside decreased eGFR. This phenomenon highlights the close relationship between diabetic nephropathy and macrovascular disease.^[32]^ Both conditions share similar pathogenic mechanisms, including inflammation, oxidative stress, and endothelial dysfunction.^[33]^ The significant correlation between TyG and cystatin C (*R* = 0.287, *P* < .001) further supports the central role of insulin resistance in renal and vascular damage.^[34]^

Stratified analyses confirmed that TyG index maintains excellent predictive value across various subgroups, particularly in individuals aged ≥ 65, with a diabetes duration < 10 years, without hypertension, and with BMI < 25 kg/m^2^. These findings have important clinical implications: the TyG index may serve as a more sensitive predictor than traditional risk factors in older adults^[35]^; it can facilitate early identification of high-risk patients with a shorter duration of diabetes^[36]^; and in normotensive patients, it may be the sole metabolic risk marker.^[37]^

Our findings are highly consistent with multiple large-scale international studies. Irace et al^[37]^ found a significant association between the TyG index and carotid intima-media thickness in an Italian population. Sánchez-Íñigo et al^[12]^ confirmed that the TyG index is an independent predictor of cardiovascular events in a Spanish cohort. However, some studies have reported racial and regional variations in the predictive utility of the TyG index.^[38]^ Research from the Asia-Pacific region generally demonstrates strong predictive performance, likely related to unique metabolic characteristics and genetic backgrounds in Asian populations.^[39]^

While the TyG index shows promise as a simple and accessible biomarker, several critical steps are required before clinical implementation can be recommended. Our single-center cross-sectional study provides preliminary evidence of association but cannot establish causality or predictive value for incident disease. Future research priorities should include: large-scale multicenter prospective studies to establish temporal relationships and validate predictive performance; external validation in diverse populations to assess generalizability; comparison with established cardiovascular risk scores to demonstrate incremental predictive value; clinical trials demonstrating that TyG index-guided interventions improve patient outcomes; and cost-effectiveness analyses to support implementation in resource-limited settings. Until these evidence gaps are addressed, the TyG index should be considered an investigational marker rather than a clinical decision tool.

This study has several limitations that need to be acknowledged. Study Design Limitations: First, this study employed a retrospective cross-sectional design, which cannot establish causality and can only determine associations between the TyG index and peripheral vascular complications rather than prove causal relationships. Second, the single-center study with a relatively small sample size (n = 186) may limit the generalizability of results, requiring larger-scale multicenter validation studies. Third, the study included only hospitalized patients, which may represent a more severely ill or unstable diabetic population, potentially limiting the generalizability of results to outpatient populations. TyG Index Measurement-Related Limitations: The use of single fasting measurements obtained during hospitalization may introduce measurement bias due to acute stress, medication adjustments, or dietary changes. Although we implemented strict exclusion criteria and standardized collection procedures to minimize these effects, residual confounding cannot be completely eliminated. We did not systematically collect data on recent pre-admission dietary patterns or document all medication adjustments during hospitalization that might affect triglyceride and glucose levels. The hospital environment may select for more severe or unstable diabetic patients, potentially limiting generalizability to outpatient populations. Future studies should consider multiple time-point measurements and include comprehensive documentation of factors that might affect acute metabolic status to strengthen the validity of TyG index assessment. Peripheral Vascular Disease Diagnostic Method Limitations: The use of composite diagnostic criteria combining functional and anatomical assessments may introduce heterogeneity in the study population, potentially affecting the generalizability of our findings to populations diagnosed using uniform criteria. The selective use of advanced imaging based on clinical indications may introduce verification bias, potentially leading to underdiagnosis of asymptomatic disease in patients with normal ABI values. However, this approach reflects standard clinical practice and resource constraints in real-world settings. The inclusion of both symptomatic and asymptomatic disease may mask important differences in pathophysiology and prognosis between these subgroups. Future studies should consider stratified analyses based on symptom status and diagnostic method to better understand these relationships. The lack of standardized assessment tools for intermittent claudication symptoms in all patients may have introduced some diagnostic variability. Medication Recording and Adjustment Limitations: A significant limitation of our study is the inadequate recording and adjustment for medications that directly affect TyG index components and peripheral vascular outcomes. While we recorded basic antidiabetic medication use (insulin and metformin), we did not systematically collect comprehensive data on: specific insulin regimens and dosages; newer antidiabetic medications with established cardiovascular benefits such as SGLT2 inhibitors and GLP-1 receptor agonists; lipid-lowering therapy including statins and fibrates that directly affect triglyceride levels; detailed antihypertensive medication categories (ACE inhibitors, ARBs, beta-blockers) that affect cardiovascular outcomes in diabetic patients; and antiplatelet therapy that affects vascular risk. This represents substantial residual confounding that may affect the observed association between TyG index and peripheral vascular complications. Differential use of these medications between patient groups may either strengthen or attenuate apparent relationships, limiting the generalizability of our findings to clinical populations with different medication regimens. Other Potential Confounding Factor Limitations: This study did not include other possible confounding factors such as inflammatory markers (C-reactive protein, interleukins), detailed lifestyle factors (smoking history, alcohol consumption, physical activity level), nutritional status assessment, and detailed stratification of other cardiovascular risk factors. These factors may independently affect peripheral vascular disease occurrence, and failure to adjust for these factors may affect result interpretation. Follow-up and Long-term Prognostic Assessment Limitations: As a cross-sectional study, we could not assess the predictive value of the TyG index for long-term prognosis or determine the relationship between dynamic changes in TyG index and disease progression. The lack of prospective follow-up data limits our evaluation of the clinical application value of the TyG index as a dynamic monitoring indicator. Despite these limitations, our study employed a reasonable study design under existing conditions, implemented strict quality control measures, and obtained clinically meaningful results. These limitations will provide important references for future research design and improvement. We recommend that future studies should implement prospective designs, multicenter collaboration, larger sample sizes, comprehensive medication recording, standardized diagnostic protocols, and long-term follow-up to overcome the limitations of this study and further validate the clinical application value of the TyG index.

In conclusion, this cross-sectional study demonstrates that the TyG index, as a simple proxy for insulin resistance, is significantly associated with peripheral vascular complications in patients with type 2 diabetes. Our findings reveal a strong cross-sectional association rather than establishing predictive capability, as the retrospective design cannot determine temporal relationships or causality between elevated TyG levels and peripheral vascular disease development. The observed associations suggest that the TyG index may serve as a useful marker for identifying patients with existing peripheral vascular complications and could potentially contribute to risk stratification in clinical settings. However, prospective longitudinal studies are essential to establish whether elevated TyG index values precede and predict the development of peripheral vascular complications, and to determine the direction of causality. While reverse causation cannot be excluded based on our study design, the biological plausibility of insulin resistance contributing to vascular pathology supports the potential utility of the TyG index in clinical assessment. Future prospective studies should validate the predictive value of the TyG index for incident peripheral vascular disease and evaluate its role in monitoring disease progression and treatment response over time.

## Author contributions

**Conceptualization:** Dong Fan, Tao Deng.

**Formal analysis:** Dong Fan, Luyao Zhang.

**Writing – original draft:** Dong Fan.

**Writing – review & editing:** Dong Fan, Tao Deng.

**Data curation:** Xiaoyu Ma.
